# Gender and Weight Shape Brain Dynamics during Food Viewing

**DOI:** 10.1371/journal.pone.0036778

**Published:** 2012-05-10

**Authors:** Ulrike Toepel, Jean-François Knebel, Julie Hudry, Johannes le Coutre, Micah M. Murray

**Affiliations:** 1 The Functional Electrical Neuroimaging Laboratory, Department of Clinical Neurosciences, Vaudois University Hospital Center and University of Lausanne, Lausanne, Switzerland; 2 The Functional Electrical Neuroimaging Laboratory, Department of Radiology, Vaudois University Hospital Center and University of Lausanne, Lausanne, Switzerland; 3 Nestlé Research Center, Vers-chez-les-Blanc, Lausanne, Switzerland; 4 The University of Tokyo, Organization for Interdisciplinary Research Projects, Tokyo, Japan; 5 EEG Brain Mapping Core, Center for Biomedical Imaging (CIBM), Lausanne and Geneva, Switzerland; University of Missouri-Kansas City, United States of America

## Abstract

Hemodynamic imaging results have associated both gender and body weight to variation in brain responses to food-related information. However, the spatio-temporal brain dynamics of gender-related and weight-wise modulations in food discrimination still remain to be elucidated. We analyzed visual evoked potentials (VEPs) while normal-weighted men (n = 12) and women (n = 12) categorized photographs of energy-dense foods and non-food kitchen utensils. VEP analyses showed that food categorization is influenced by gender as early as 170 ms after image onset. Moreover, the female VEP pattern to food categorization co-varied with participants' body weight. Estimations of the neural generator activity over the time interval of VEP modulations (i.e. by means of a distributed linear inverse solution [LAURA]) revealed alterations in prefrontal and temporo-parietal source activity as a function of image category and participants' gender. However, only neural source activity for female responses during food viewing was negatively correlated with body-mass index (BMI) over the respective time interval. Women showed decreased neural source activity particularly in ventral prefrontal brain regions when viewing food, but not non-food objects, while no such associations were apparent in male responses to food and non-food viewing. Our study thus indicates that gender influences are already apparent during initial stages of food-related object categorization, with small variations in body weight modulating electrophysiological responses especially in women and in brain areas implicated in food reward valuation and intake control. These findings extend recent reports on prefrontal reward and control circuit responsiveness to food cues and the potential role of this reactivity pattern in the susceptibility to weight gain.

## Introduction

The prevalence of eating disorders and, in particular, overweight is increasing throughout the world population causing long-term health problems such as diabetes or cardiac disorders. Obesity is linked to a loss in the ability to adjust food intake for maintaining the energetic balance of the body. Although both genders are affected, women have been found to be more susceptible to developing eating disorders [1] and also obesity [2]. The biological bases for these gender differences in normal-weighted individuals are currently being investigated, with hormonal and cerebral variation representing likely candidate factors (e.g. [3], [4]). The present study focuses on spatio-temporal brain dynamics of gender differences in the visual processing of food images and their correlation with body-mass index (BMI). Given evidence for the rapid interplay between visual and reward-related brain activity (e.g. [5]), visual evoked potentials and their millisecond temporal resolution were combined with distributed source estimations to determine the primary loci of gender differences. Comparing food and non-food image processing likewise allowed for the determination of any specificity in such gender differences. Finally, inclusion of BMI as a covariate in our analyses would allow for a degree of inference regarding the potential relation between the consequences of food intake behavior (which we assume to be quantified in a gross and cumulative sense by BMI) and modulations in brain activity to specific classes of stimuli.

Differences in food perception between genders (and also between normal- vs. over- weight populations) have so far mainly been investigated with hemodynamic neuroimaging. DelParigi and colleagues [6] measured regional cerebral blood flow (rCBF) at rest in normal-weight women and men after a 36 h-fast and after subsequent satiation with a liquid meal. Despite largely overlapping activations in cortical and subcortical frontal as well as temporal regions, women showed stronger responses than men in both parieto-occipital and dorsolateral prefrontal cortices after satiation. Male responses were, on the other hand, stronger than female ones in the ventromedial prefrontal cortex under hungry conditions. Associations between hemodynamic responses and gender have further been investigated during the sensory perception of gustatory and visual food stimuli [7], [8] revealing elevated responses in women as compared to men, particularly in frontal brain areas. Along these lines, Killgore and Yurgelun-Todd [9] found gender differences when women and men differentiated images of high- and low-calorie foods, with women especially showing enhanced activity to high-caloric foods in ventral and dorsal prefrontal regions. These findings likely indicate a varying motivation towards food intake between genders and differential behavioral control mechanisms in women as opposed to men.

Apart from gender, body weight (as quantified by BMI) was found to be an additional factor influencing cerebral responsiveness to food viewing even in normal-weight women. Killgore and Yurgelun-Todd [10] reported negative correlations between activity in prefrontal regions and BMI, such that increases in BMI were associated with reduced frontal activity. These findings have been linked to a decisive role of prefrontal brain areas in the mediation of food motivation and control over food intake [6], [9], [10], [11], [12], [13], pointing out their relevance in relation to deviant eating styles and prospective weight gain. Taken together, hemodynamic imaging revealed associations between gender and even small variations in BMI on brain processes involved in food perception. So far, the temporal dynamics of these processes remain largely unknown, although they could prove to be important for the development of targeted cognitive-behavioral weight management strategies in the long run [14], [15]. Plus, the timing of gender-related differences may be linked to the specific class of stimuli employed. Previous VEP studies using other types of biologically salient stimuli other than food (such as faces or emotional pictures) present rather mixed evidence as to the timing of gender-driven VEP modulations. Some studies revealed gender influences on the VEP amplitudes between 100–200 ms post-stimulus onset [16], [17], [18], whereas others found only later modulations ∼300–400 ms post-stimulus onset [19], [20]. Differences across studies may alternatively be linked to ambiguity stemming from the statistical analyses employed, which motivated our use of multivariate and reference-independent metrics here [21], [22].

Female socialization often results in stronger preoccupation with nutritional aspects and body image [23], [24] likely influencing pre-ingestive visually-based food choices as well. Thus, we expected modulations of VEP patterns and neural source activity during food categorization to be influenced by gender within similar time ranges as in the abovementioned VEP studies and investigations on the time course of food discrimination [5], [25]. Moreover, we expected that the electrophysiological responses would be associated with weight (i.e. BMI) in women even when being within limits considered as normal. Whether men would reveal similar relations between brain responses to food viewing and BMI remained elusive.

## Materials and Methods

### Participants

Twenty-four (12 female) remunerated volunteers, aged 19–36 years (mean±s.e.m. = 25.8±1.1 yrs), participated in the study [5]. Nineteen of these participants were right-handed, and five were ambidextrous according to the Edinburgh Handedness Inventory [26]. Their BMIs were within normal limits (women = 19–24 kg/m^2^, mean = 20.67 [s.e.m. ±0.54]; men = 20–25 kg/m^2^, mean = 23.0 [s.e.m. ±0.63]), the BMI range being similar between genders. None of the participants reported current or prior neurological or psychiatric illnesses or a history of dieting or eating disorders. All participants had normal or corrected-to-normal vision. All of the EEG recording sessions started between 13:00h–14:00h to control for circadian modulations of hunger. Further, participants were instructed to have eaten lunch to satiety before the recording sessions. They likewise explicitly reported what they had eaten for both breakfast and lunch that day in a questionnaire. All participants provided written, informed consent to the procedures, which were approved by the Ethics Committee of the Faculty of Biology and Medicine of the University of Lausanne and the Vaudois University Hospital Center (CHUV).

### Stimuli and procedure

**Figure 1:**
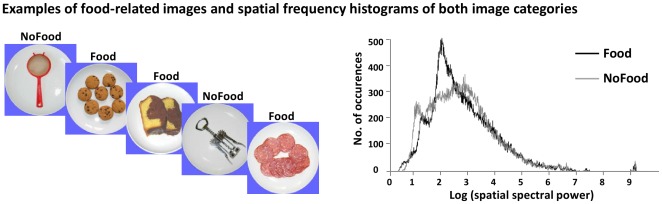
Exemplar photographs of foods and non-food kitchen utensils (left panel) and spatial frequency distributions of each image category (right panel).

Participants were presented with four blocks of trials each comprising 50 non-complex colorful photographs of energy-dense high-fat foods and 50 non-food kitchen utensils (see [5]) in pseudo-randomized orders (Figure 1). A questionnaire presented to 10 women and 10 men assured that images classes were equally familiar to both genders (no interaction of image category and gender). The luminosity of the images had been normalized [27], but the spatial frequencies between categories could not be fully matched (see Figure 1) [5]. We would remind the reader, however, that the main objective of this study was to assess interactions between gender and image category, rather than to identify main effects of image category. All photographs measured 300×300 pixels, which corresponded to ∼6° visual angle on the computer monitor and were taken using an identical background from an identical top-view angle. For data acquisition, images were centrally presented for 500 ms on a 21″ CRT monitor that participants viewed within an electrically shielded and sound-attenuated booth. Immediately after each image a question mark was presented, indicating that participants should indicate via button-press if the preceding image had been a food or a non-food item. This was done to separate in time visual and perceptual processes from motor-related potentials. One downside is that reaction time data are less informative about the underlying perceptual/cognitive operations performed by participants. To minimize eye movements, a central fixation cross was present whenever no image or question mark was present. Following response execution, the inter-trial interval (ITI) varied randomly from 250–750 ms. Stimulus presentation and response recordings were controlled by E-Prime (Psychology Software Tools Inc., Pittsburgh, USA; www.pstnet.com/eprime).

### Electroencephalography (EEG) acquisition and preprocessing

Continuous EEG was acquired at 512 Hz though a 160-channel Biosemi ActiveTwo system (Biosemi, Amsterdam, Netherlands) referenced to a ground circuitry (common mode sense and driven right leg electrodes or CMS-DRL, respectively). This circuitry functions as a feedback loop driving the average potential across the montage as close as possible to the amplifier zero. Details of this circuitry, including a diagram can be found on the Biosemi website (http://www.biosemi.com/pics/zero_ref1_big.gif). All analyses were conducted using the CarTool software (http://sites.google.com/site/fbmlab/cartool) and the Sten toolbox (www.unil.ch/fenl/Sten). To calculate VEPs, epochs of EEG from 98 ms pre- to 488 ms post- stimulus onset (i.e. 50 data points before and 250 data points after stimulus onset) were separately averaged for each image category and each participant. In addition to an automatic ±80 µV artifact rejection criterion, EEG epochs containing eye blinks or other noise transients were removed by trial-to-trial inspection of the data. Prior to EEG epoch averaging as a function of gender and image category, data from artifact electrodes of each participant were interpolated using 3-D splines [28]. Data were band-pass filtered (0.01–40 Hz), baseline corrected using the 98 ms pre-stimulus period, and recalculated against the average reference. The number of accepted VEP epochs for food and non-food viewing did not differ between genders. Moreover, to account for potentially different skull conductivity between genders, all epochs were normalized by mean Global Field Power (GFP) at each time point [29]. GFP is calculated as the square root of the mean of the squared value recorded at each electrode in the 160-channel montage (vs. the average reference) and represents the spatial standard deviation of the electric field at the scalp [30].

### EEG Analyses and source estimation

#### General analysis strategy

VEP analyses were applied using both global and local measures of the electric field recorded from 160 scalp surface sensors. These so-called electrical neuroimaging analyses allow differentiating effects caused by modulations in the strength of responses of statistically indistinguishable brain generators from alterations in the generator configuration (i.e. the topography of the electric field). We provide only the essential details on the methods here (but see [21], [31]. ANOVAs included the within-subject factor image category (food and non-food) and the between-subject factor gender, and focused on detecting interactions between these factors. In order to avoid confounds from the dissimilar spatial frequency power between image categories (see Figure 1) we refrain from reporting simple effects of image category. Temporal autocorrelation at electrode sensors was controlled for by considering only those effects reliable where p-values exceeded the statistical threshold (p≤0.05) for more than 15 contiguous time points (∼30 ms; [32]).

#### Global and local VEP Analyses

In order to objectively determine stable VEP intervals that reveal response modulations as a function of image category and gender, the collective post-stimulus group-average VEP periods were subjected to a topographic cluster (i.e. VEP map) analysis based on a hierarchical clustering algorithm [21]. The clustering serves to subsume stable electric field topographies to periods of stable VEP topographies or maps. VEP topography is independent of the reference and modulations in topography forcibly reflect modulations in the configuration of underlying generators [33]. Additionally, the clustering is exclusively sensitive to topographic modulations because the data are first normalized by their instantaneous GFP. The optimal number of stable VEP clusters (i.e. the minimal number of maps that accounts for the greatest variance of the dataset) was determined using a modified Krzanowski-Lai criterion [21]. The clustering makes no assumption on the orthogonality of the derived topographic template maps [34], [35]. Stable group-level VEP cluster periods over which differential map patterns were observed entered a fitting procedure. This fitting labeled each time point of each single subject VEP according to the group-average topographic map it best correlated with spatially [21], so as to statistically test the presence of a each map in the moment-by-moment scalp topography of individuals' VEPs. The dependent measure derived is the global explained variance (GEV) of the map identified in the group-average data in the response of an individual participant and each condition. GEV is measured as the sum of the explained variance (EV) as a function of time, weighted by the GFP at each moment in time. Likewise, the EV at a given moment in time equals the squared value of the spatial correlation between a given template map and the single-subject VEP. These values were submitted to repeated measures ANOVA with the within-subject factors of image category and map and the between-subject factor of gender. The fitted map with the highest GEV over a given time period best describes the VEP during this period.

We further computed VEP means for each participant, condition and electrode sensor over the time interval of stable VEP clustering (here: 170–213 ms). An electrode-wise ANOVA with the within-subject factor of image category (food and non-food) and the between-subject factor of gender then served to visualize local VEP interactions between factors. The result of this analysis is presented as an intensity plot representing time, electrode location, and significant p-values. We would like to stress, however, that our main conclusions are based on global measures of the electric field, i.e. incorporating data points from all electrode sensors in a conjoined and reference-independent manner.

Modulations in the strength of the electric field at the scalp were assessed by a time point-wise ANOVA on the Global Field Power (GFP; [29]) with the factors of image category and gender. GFP yields larger values for stronger scalp-electric fields. As the GFP analysis is independent and orthogonal to topographic measures on the electric field, it can thus be observed whether topographic changes are accompanied by global VEP amplitude modulations.

#### Source Estimations

Intracranial sources underlying the surface electric fields were estimated for each image category and gender using the local auto-regressive average (LAURA) distributed linear inverse solution [36], [37]. This algorithm selects the source configuration based on the biophysical behavior of electric vector fields. The version of LAURA used here employs a realistic head model with 3005 nodes arranged within the gray matter of the Montreal Neurological Institute's (MNI) average brain. This implementation of LAURA was generated with the Spherical Model with Anatomical Constraints (SMAC; [38]). As an output, LAURA provides current density value (in µA/mm^3^) at each node. Prior fundamental and clinical research have documented and discussed in detail the spatial accuracy of this inverse solution, which are on the order of the grid size of the solution points (here ∼6×6×6 mm; [31], [39], [40]). The time period for which intracranial sources were estimated and statistically compared between conditions was defined by the results of the abovementioned topographic cluster analysis (here: 170–213 ms). Statistics on the source estimations was performed by first averaging the VEP data over the 170–213 ms interval to generate a single data point for each participant to increase the signal-to-noise ratio. The inverse solution (12 participants×2 genders×2 categories) was then estimated for each of the 3005 nodes. An ANOVA was performed with the within-subject factor of category (food vs. non-food) and the between-subject factor of gender (women vs. men) at each source node. Only effects corrected for false discovery rate (FDR) and present in ≥15 contiguous nodes were considered significant. The latter spatial criterion was determined using the AlphaSim program (http://afni.nihm.nih.gov) as in former reports from our group [5], [35], [41]. The source nodes showing a significant interaction between the factors image category and gender were rendered on the MNI average brain for visualization.

Further, two-tailed Pearson correlations between the current density value (in µA/mm^3^) of each source node and to each image category (food and non-food) with the respective BMI of individuals were calculated for each gender separately. Only nodes showing reliable correlations (r_(10)_±0.58; p≤0.05) and exceeding the spatial criterion of ≥15 contiguous nodes were considered significant. In addition, the interaction term obtained by the abovementioned ANOVA served to restrict the obtained correlations to brain regions in which source node activity significantly differed as a function of the viewed image category and gender. The results of the correlation analyses were rendered on the MNI brain with the Talairach and Tournoux coordinates [42] of the correlation maxima indicated.

## Results

The 24 participants readily performed the discrimination between images of foods and kitchen utensils. Women responded correctly to 96.79% (s.e.m. ±0.86) of the food images and to 95.41% (s.e.m. ±1.14) of the non-food images. Men answered correctly to 96.54% (s.e.m. ±0.96) of the foods and to 95.27% (s.e.m. ±1.50) of the non-foods. An ANOVA with the within-subject factor image category and the between-subject factor gender revealed an effect of category (F_(1,22)_ = 4.95; p≤0.05), but no main effect of gender or interaction between category and gender.

Women responded with a mean (±s.e.m.) reaction time of 406±27 ms to food images and 422±25 ms to non-food images. Men responded with a mean (±s.e.m.) reaction time of 309±26 ms to food images and 291±24 ms to non-food images. The 2×2 ANOVA revealed an interaction between category and gender (F_(1,22)_ = 5.62; p≤0.05). Post-hoc unpaired t-tests between genders showed that men were reliably faster than women in categorizing the food images (t_(22)_ = 2.62; p≤0.05) as well as the non-food images (t_(22)_ = 3.81; p≤0.01). We would remind the reader, however, that reaction times were cued by a question mark that appeared subsequent to image presentation so as to avoid contamination of the VEP by motor-related activity. Thus, the above reaction times likely do not reflect the true latency of decision-related processes. We nonetheless include them here for completion.

**Figure 2:**
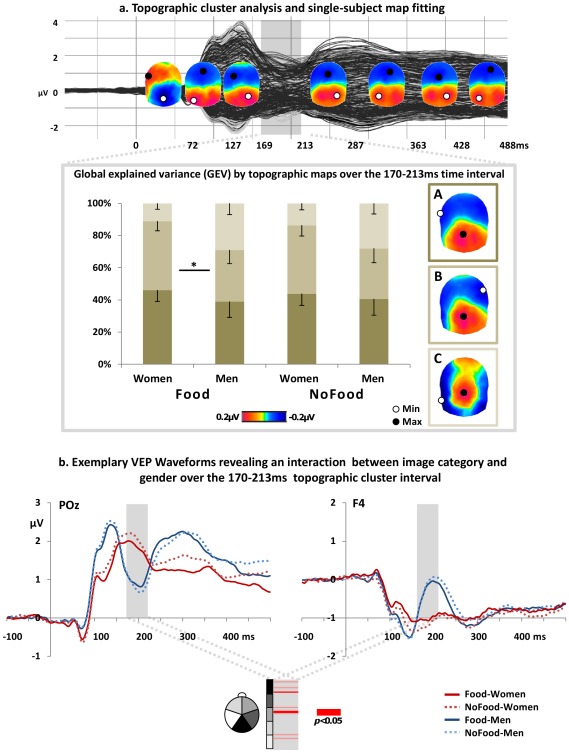
Results of VEP analyses. (a) A topographic cluster analysis incorporating the group-average VEPs of both genders to both image categories (food and non-food) revealed eight VEP cluster periods. Statistics on the (back-)fitting of group-average topography to single subject VEPs revealed that topographic map presence (as measured by global explained variance GEV) was modulated by gender and image category over the 170–213 ms interval after image onset. Bar graphs illustrate the GEV (± s.e.m.) of each topography present over the 170–213 ms interval (i.e. map A, B or C) in the responses of women and men when viewing images of food and non-food. The asterisk illustrates significant differences between the presence of map B between female and male responses to food as obtained from post-hoc t-tests. (b) Exemplar VEP waveforms from both genders in response to food- and non-food viewing at a posterior parietal (POz) and a right frontal electrode (F4). Below, a p-value plot illustrates over which scalp region electrode sensors ( = 160) revealed a reliable interaction between image category and gender over the 170–213 ms interval.

As illustrated in the top panel of Figure 2a, the VEP topographic cluster analysis identified eight stable periods over the post-stimulus epoch that explained 98.82% of variance in the collective dataset. While map topographies were similar between image categories and genders over most of the post-stimulus period, the interval from 170–213 ms yielded three differing map topographies at the group-average VEP level (termed A, B and C in the lower panel of Figure 2a). Therein, the spatial correlation between Map A and B was as high as 96%, equaled 30% between Map A and C, and was 44% between Map B and C. For the statistical validation of the topographic patterning, we obtained the spatial correlation of the group-level responses with the individual subject responses for each data sampling point over the 170–213 ms interval. The output of this map fitting procedure is the global explained variance in percent (GEV; see Methods section). The bar chart in Figure 2a visualizes the distribution of each topographic map as a function of gender and image category.

We conducted an ANCOVA on these GEV values with the within-subject factors image category and map, the between-subject factor gender and the covariate BMI. All reported values were adapted by Huynh-Feldt corrections. The ANCOVA revealed an effect of category (F_(1,20)_ = 9.61; p≤0.01; η^2^ = 0.32) as well as interactions of category×gender (F_(1,20)_ = 10.04; p≤0.01; η^2^ = 0.33), category×BMI (F_(1,20)_ = 9.22; p≤0.01; η^2^ = 0.32), category×map (F_(2,40)_ = 3.75; p≤0.05; η^2^ = 0.16), category×gender×BMI (F_(1,20)_ = 10.32; p≤0.01; η^2^ = 0.34) and category×map×BMI (F_(2,40)_ = 3.58; p≤0.05; η^2^ = 0.15). A marginally significant interaction was furthermore found between category×map×gender (F_(2,40)_ = 3.47; p = 0.057; η^2^ = 0.15). These results indicate that the VEP pattern over the 170–213 ms interval is modulated by gender and BMI and also by whether participants viewed food or non-food objects.

Separate ANCOVAs for each gender revealed an effect of category (F_(1,10)_ = 21.60; p≤0.01; η^2^ = 0.68) as well as interactions of category×BMI (F_(1,10)_ = 19.67; p≤0.01; η^2^ = 0.66), category×map (F_(2,20)_ = 7.24; p≤0.01; η^2^ = 0.42) and category×map×BMI (F_(2,20)_ = 6.13; p≤0.05; η^2^ = 0.38) in women only. In men, no effects or interactions were observed (all F<1). In summary, these results show that the modulation in VEP topography over the 170–213 ms interval is associated with BMI when women, but not men, discriminate food from non-food.

As a complement to the topographic analyses, we further conducted a sample point-wise 2×2 ANOVA on GFP, a global measure of response strength that is orthogonal to the variations in VEP response topography (see Methods section). This ANOVA revealed no time intervals of gender effects or interactions between image category and gender. This latter finding argues against a simple explanation based on heightened arousal/attention in women vs. men, which would have resulted in stronger VEPs (particularly during early processing stages).

In addition to the global VEP analyses, Figure 2b provides an illustration of the local VEP waveform pattern for each image category and gender at two exemplar electrode sensors as well as the interaction effect between image category and gender obtained by the electrode-wise ANOVA over the 170–213 ms period. As apparent from the p-value plot for the 160 electrode sensors, most pronounced interactions between both factors were apparent over parieto-occipital and right frontal electrode locations.

To this point, our analyses indicate that VEPs in men and women resulted in stable but differing response topographies between 170–213 ms post-stimulus onset when categorizing food and non-food images. Furthermore, the female response pattern during food viewing co-varied with BMI despite the fact that participants' weight was within normal ranges.

**Figure 3:**
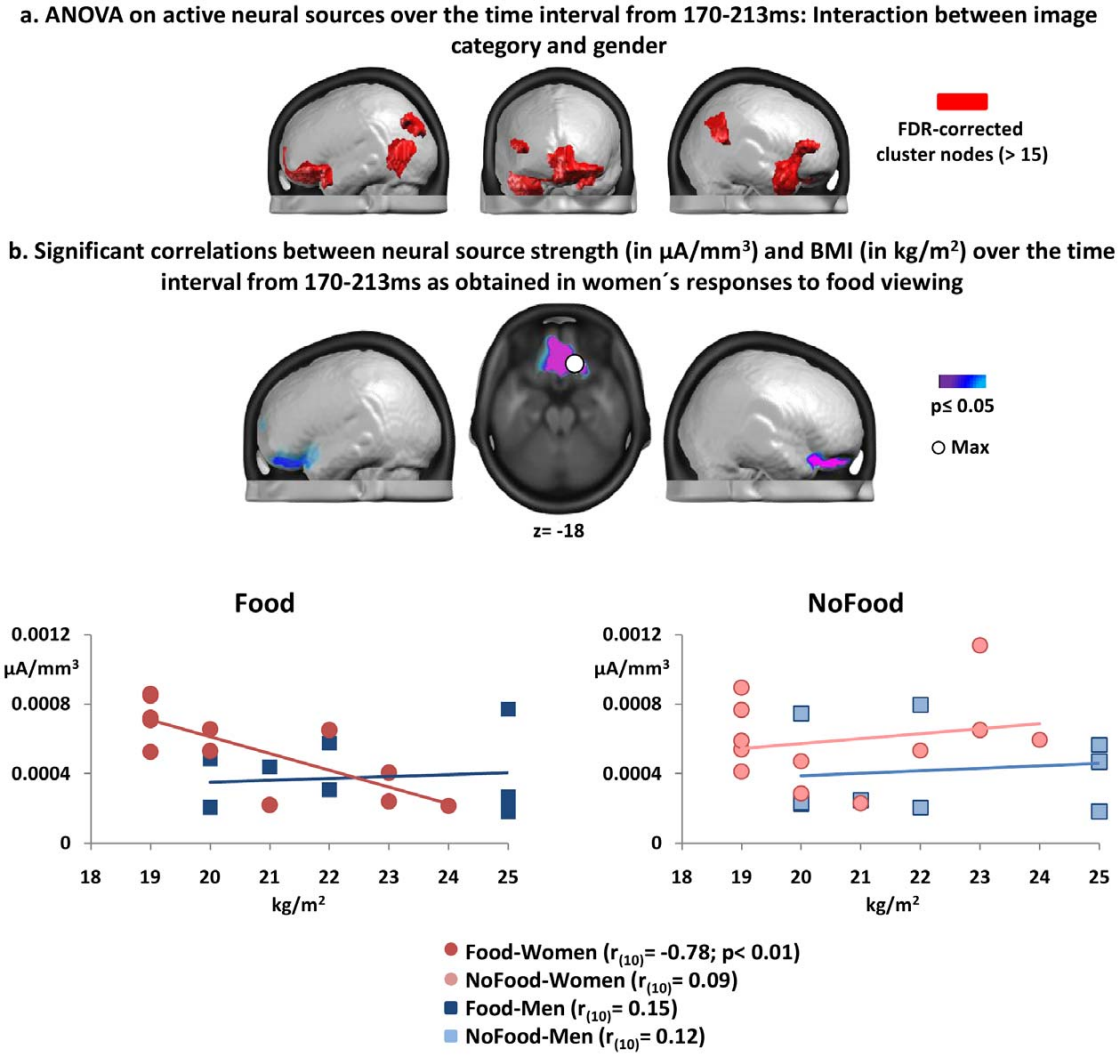
Results of the statistical analyses on neural source estimations rendered on the MNI template brain. (a) Brain regions revealing interactions between category and gender over the 170–213 ms interval. (b) A ventral prefrontal cluster yielded significant correlations between source node activation strength and BMI for female food viewing responses rendered. The scatter-plots detail the relation between the source node amplitude of female and male individuals (y-axis) and BMI (x-axis) to the viewing of each image category at the cluster maximum.

As topographic modulations are indicative of changes in the neural generator activity underlying the surface VEP pattern, we estimated the active neural sources in women and men over the time interval showing topographic variations (i.e. 170–213 ms post-image onset) when viewing either image category (see figure S1 for an illustration of the extended neural networks activated by image viewing in each gender). The source node strengths were statistically compared in an ANOVA (see Methods section) and source node clusters revealing an interaction between image category and gender were rendered on the MNI average brain for visualization (Figure 3a). Significant interactions between category and gender were obtained in source node clusters located in the ventral prefrontal cortex (Max: −22, 45, −13; BA 11), the posterior middle temporal cortex (Max: −48, −61, 11; BA 22/37) and the superior parietal lobe (Max: −28, −70, 47; BA 7) of the left hemisphere. In the right hemisphere, the interaction effects were observed in the ventromedial prefrontal cortex (Max: 3, 44, −7; BA 10), in the anterior temporal cortex (Max: 49, 10, −20; BA 38) and in the inferior parietal lobe (Max: 50, −56, 45; BA 40).

In succession, we calculated correlations between all source nodes showing the interaction effect and individuals' BMI. Significant (negative) correlations between neural source strength and BMI were only observed in the female responses to food viewing (Figure 3b), restricted to the ventromedial prefrontal cortex (Max: 16, 26, −18; BA 11). That is, neural activity in this region decreased with increasing BMI when women viewed images of energy-dense foods. Scatter-plots detail the correlations for both categories and genders at the cluster maximum and its immediate neighbors. We would like to note that non-parametric correlations for the same source node locations confirmed the observed pattern in female food viewing responses (ρ_(10)_ = −0.80; p<0.01). Furthermore, even when excluding the responses of female participants with lowest BMIs (i.e. below 20 kg/m^2^), the negative correlation remained reliable (p<0.05 for both parametric and non-parametric testing).

## Discussion

In the current study, we investigated whether gender and variations in BMI within the normal range influence the spatio-temporal brain dynamics in the visual categorization of food-related objects. We found that gender and BMI both modulate the topography of VEPs and neural source activity over the time interval from 170–213 ms after image onset. However, only in women the VEP topography during food – non-food discrimination and neural source strength in ventromedial prefrontal cortex when viewing food objects was associated with BMI over the derived time period.

The obtained time course of gender influences in our study coincides with previous findings on gender differences between ∼100–200 ms when biologically relevant objects such as faces [16] and affective stimuli [17], [18] are discriminated. Moreover, these gender influences are apparent at similar latencies at which other VEP studies observed initial food-related categorization effects, i.e. ∼150–200 ms [5], [25]. That is, gender seems to implicitly influence the discrimination of food-related objects.

Further, we found evidence that the global VEP topography during food-related object categorization co-varies with BMI in normal-weight women, but not in men, over an interval from 170–213 ms after image onset. On the other hand, we did not observe interactions between image category and gender in global VEP strength (i.e. GFP) in our group of normal-weight subjects. That is, interactions between image category and gender were more apparent in qualitative than in quantitative analyses of the VEP topography. These findings thus complement previous VEP studies that focused more on local differences in response amplitudes between normal- and overweight subjects during food-related object perception without special regard to gender variations [43], [44].

In accordance with the topographic pattern observed over the 170–213 ms time interval, we also observed interactions between image category and gender in neural source activation strength in frontal and temporo-parietal regions. These areas have previously been implicated in food discrimination at a similar timing [5] without regarding gender differences. Within those regions, we obtained inverse associations between neural source strength and BMI in female responses to food viewing; activation of the ventromedial prefrontal cortex (PFC) decreased with higher BMI in women despite of being within normal weight limits. No such associations were observed for male responses to food viewing or in either gender in response to the viewing of non-food objects.

Associations between weight and brain activation patterns have hitherto been shown by several hemodynamic imaging studies comparing brain responses between normal-weight and obese subjects during food viewing [11], [12], [45], [46]. The studies reported differential hemodynamic responses in obese as opposed to normal-weighted subjects in multiple areas, i.e. ventral and dorsal PFC as well as temporal and parietal regions, which were generally attributed to an increased motivation towards food intake as well as altered mechanisms towards food intake control and self-monitoring in the obese. Further, a study by Rothemund et al. [45] obtained positive associations between the hemodynamic response to high-energy foods and BMI in striatal regions and ventral PFC when considering data from women ranging from normal-weight to overweight. Yet, when restricting the correlation analysis to normal-weight women only, they observed inverse associations between neural activity and BMI in the ventral PFC. This finding is also in line with results of an fMRI study that reported reduced hemodynamic responses to visual food cues with increasing BMI in the ventral PFC of normal-weight women [10].

The ventral PFC is an area in which several sensory, cognitive and affective inputs converge, and signals are computed that guide behavioral goal-directed values and choices [47], [48], [49], [50]. With respect to food perception, the role of the PFC in food perception has been directly linked to reward valuation and control mechanisms over food intake [6], [9], [13], [46], [47], [50], [51], [52], [53], [54]. There is ongoing debate concerning associations between PFC (and striatal) activation patterns and weight measures in terms of the role of hypo- vs. hyper-responsiveness of these regions in obesity and weight gain prediction. Therein, hemodynamic studies revealed general patterns of hyper-responsivity of food reward circuits in overweight subjects [45], [46], [55]. At the same time, when using prefrontal and striatal activation during food perception to predict prospective weight gain, hypo-responsivity of these structures has been shown to relate to later increases in BMI [55], [56]. Since the present study applied a distributed source estimation approach, we would nonetheless be remiss if we did not mention that limitations remain concerning the ability to reliably detect sources within deep structures such as the striatum. Thus, we cannot wholly exclude the possibility of additional sources contributing to our effects.

Yet, our study also points to a decreased responsiveness of prefrontal regions implicated in reward evaluation and food intake control with increasing weight, but only in women. The time course of the obtained association in women indicates that neural response attenuation is already effective within 200 ms after food cue onset, i.e. over a time interval showing initial food categorization effects modulated by viewer's gender. That is, varying food cue responsiveness between genders as a function of weight may present an indication towards an elevated vulnerability of women to potential weight gain. As most studies have hitherto addressed this issue in women only, prospective studies on the relation between responses of food reward/control circuits and weight gain in both genders are needed to further elucidate gender influences on weight gain susceptibility. Furthermore, previous reports evinced an elevated responsiveness of prefrontal and striatal regions in overweight participants, potentially indicating a switch in food reward and control mechanisms along a weight continuum that might mediate a change in eating style from a more ‘cognitive’ reflective mode to a more ‘automatic’ reflexive mode [54]. Designating such a switching point in more detail in the future could be an asset to develop cognitive-behavioral intervention strategies targeting deviant eating styles leading to overweight. One important limitation in the present study is, however, that standardized questionnaires were not completed by our participants, making it possibly that there is an under-estimation of, e.g., restrained eating behavior that in turn may (partially) contribute to our pattern of results. Likewise, because only participants with BMI within the normal-range partook in the current study, longitudinal data on spatio-temporal brain dynamics in relation to weight gain patterns are so far not available.

## Supporting Information

Figure S1 **Results of the neural source estimations over the 170–213 ms interval during food viewing (upper panels) and non-food viewing (lower panels) in women (left panels) and men (right panels) rendered on the MNI template brain.** In both genders, viewing energy-dense foods addressed temporal, parietal and occipital regions in both hemispheres. In women, the active neural sources were more widely distributed than in men, also comprising inferior prefrontal areas. Neural sources during the viewing non-food images in both genders were less disseminated than during food viewing. Results on the statistical differences in activation patterns as a function of image category and gender are shown in Figure 3a.
(TIF)
