# Supplementary figures and images for: Gender and Weight Shape Brain Dynamics during Food Viewing
Source: PLoS One. 2012 May 10;7(5):e36778. doi: 10.1371/journal.pone.0036778 (PMC3349646; doi:10.1371/journal.pone.0036778)

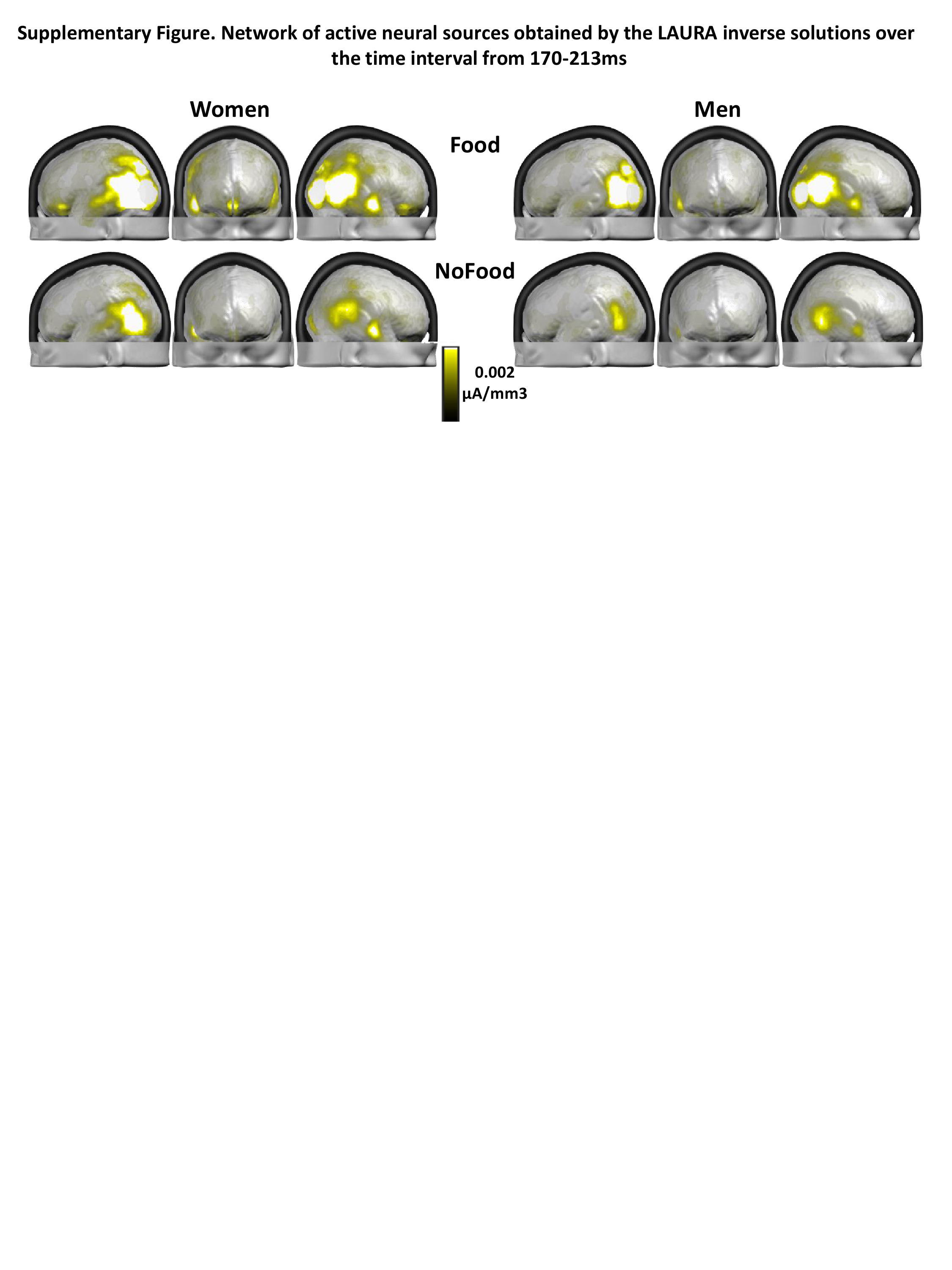

Supplement: Figure S1 — Results of the neural source estimations over the 170–213 ms interval during food viewing (upper panels) and non-food viewing (lower panels) in women (left panels) and men (right panels) rendered on the MNI template brain. In both genders, viewing energy-dense foods addressed temporal, parietal and occipital regions in both hemispheres. In women, the active neural sources were more widely distributed than in men, also comprising inferior prefrontal areas. Neural sources during the viewing non-food images in both genders were less disseminated than during food viewing. Results on the statistical differences in activation patterns as a function of image category and gender are shown in Figure 3a. (TIF) [file pone.0036778.s001.tif]
